# Macrophage-derived long non-coding RNAs in cancer: pioneering targets for immune modulation and personalized therapy

**DOI:** 10.3389/fimmu.2026.1711686

**Published:** 2026-04-28

**Authors:** Prasanna Srinivasan Ramalingam, Md Sadique Hussain, Yumna Khan, Mudasir Maqbool, Mohit Agrawal, Haleema Shahin Dh, Ashwini Prabhu, Sumel Ashique, Jyoti Maithani Kalra, Kapil Kalra, Purushothaman Balakrishnan, Sivakumar Arumugam

**Affiliations:** 1 Protein Engineering Lab, School of Biosciences and Technology, Vellore Institute of Technology, Vellore, Tamil Nadu, India; 2 Uttaranchal Institute of Pharmaceutical Sciences, Uttaranchal University, Dehradun, Uttarakhand, India; 3 Independent Researcher, Abu Dhabi, United Arab Emirates; 4 Department of Pharmacology, Government Medical College Baramulla, Jammu and Kashmir, Baramulla, India; 5 Department of Pharmacology, School of Medical & Allied Sciences, K.R. Mangalam University, Gurugram, India; 6 Department of Pharmacology, Yenepoya Pharmacy College & Research Centre, Yenepoya (Deemed to be University), Mangalore, Karnataka, India; 7 Division of Cell and Molecular Biology, Central Research Laboratory, Srinivas University, Mangalore, India; 8 Department of Pharmaceutical Technology, Bharat Technology, Howrah, West Bengal, India; 9 School of Pharmaceutical Sciences, Shri Guru Ram Rai University, Dehradun, Uttarakhand, India; 10 Alpine College of Management and Technology, Dehradun, Uttarakhand, India; 11 TanBio R and D Solution, Thiruvarur, Tamil Nadu, India

**Keywords:** immune modulation, immunotherapy, macrophages, personalized medicine, tumor microenvironment, tumor-associated macrophages

## Abstract

Generated from macrophages, long non-coding RNAs (lncRNAs) are increasingly recognized as imperative in influencing cancer immune evasion, microenvironment alteration, and therapy sensitivity. Together with tumor-associated macrophages (TAMs), this detailed investigation investigates the several roles of lncRNAs derived from M1 and M2 macrophage subtypes in regulating tumor progression. In contrast to the cancer-promoting effect of M2-based derived lncRNAs, such as H19, AFAP1-AS1, and CRNDE, which stimulate tumor proliferation, metastases, and chemoresistance through a variety of cascades, M1-based derived lncRNAs, such as HOTTIP and NBR2, exhibit tumor-suppressive roles in augmenting inflammatory signals and inhibiting epithelial-mesenchymal transition. Other than their involvement in the development of cancer, macrophage-derived lncRNAs have important immune control effects based on macrophage polarization, cytokine production, and immune checkpoints. Recent progress in single-cell RNA sequencing and the CRISPR-based functional studies also now provides new lncRNA targets and improved accuracy of diagnostics and optimization of therapy approaches. It marks a new step in personalized cancer immunotherapy: our research focuses on the possibilities of macrophage-derived lncRNAs as biomarkers and treatment options.

## 1 Introduction

Cancer growth is characterized by genetic mutations, alteration of the epigenetic profile, and communication with the environment, all of which are dynamic, multistage. The immune cell, stromal component, blood vessel, and extracellular matrix tumor microenvironment (TME) have a great impact on tumor formation (1). Besides offering a mechanical support regime, the TME also encourages tumor growth, angiogenesis, immune homeostasis, and metastases. Recent developments also indicate that TME-responsive therapeutic platforms, such as nanotechnology-based and multimodal platforms (2), are capable of actively remodeling the TME and enhancing therapeutic efficacy, especially when combined with immunotherapy strategies (3, 4). The past few years have provided compelling evidence of the interplay between tumor cells and the TME because interest has been growing in recent years (5, 6). Tumor-associated macrophages (TAMs) are rather necessary and may assume a large range of polarization forms, which can be generally characterized as either M1 or M2, these two extremes of a broad continuum of polarization states of the different parts of the TME (7). M1-type macrophages are inflammatory agents with antitumorigenic potentials, which release the TNF-α and interleukin-12 (IL-12) (8). IL-10- and vascular endothelial growth factor (VEGF)-induced M2-like macrophages lead to tumor progression by immunosuppression, stimulating angiogenesis, and stimulating matrix remodeling (9). By accentuating their relevance as possible therapeutic biomarkers, M2-like TAMs composition in the TME has been tied to adverse clinical outcomes and treatment resistance (9, 10).

The members of the lncRNA transcripts above 200 nucleotides in size include a myriad of components. LncRNAs were initially viewed as transcriptional noise, but studies have since identified that these RNAs are key regulators of gene expression at both ends of transcription and also in epigenetics and post-transcriptional processes (11). Cancer research has also implicated lncRNAs’ involvement in such activities as cell growth, intracellular spreading, and disease diffusion outside of the site of initial location and resistance to drugs developed during treatment (12). Furthermore, the results show that lncRNAs are pertinent in the regulation of TME and even the macrophage polarization (13). The therapeutic intervention of lncRNAs is also an interesting concept with capabilities to change the TME by means of signaling pathways and immunological responses modification (14, 15).

In our opinion, the key significance of macrophage-derived lncRNAs is that they can bridge tumor-induced environmental cues and sustainable macrophage functional conditions. We do not view these molecules as biomarkers only of macrophage activation, but as regulators of macrophage polarization: as transcriptional, post-transcriptional, and epigenetic regulators. This is of great importance in cancer as hypoxia, cytokines, metabolites, and extracellular vesicle (EV)-mediated signals in the TME continuously shape tumor-associated macrophages. Thereby, macrophage-derived lncRNAs can be regarded as tumor-immune communication integrators, whose direct effects are on immune suppression, metastatic progression, and responses to therapy.

Since the research question is based on the recent role of macrophage-derived lncRNAs in tumor biology, the present review will critically analyze how these regulate macrophage polarization, remodel tumor microenvironment, and control cancer progression and cancer therapeutic response. In particular, the article will answer the following three key questions: (i) how macrophage-derived lncRNAs promote the functional plasticity of macrophages, such as M1, M2, and tumor-associated macrophage phenotypes; (ii) how these lncRNAs influence immune control, tumor development, metastasis, and immunotherapeutic resistance; and (iii) whether macrophage-derived lncRNAs may serve as clinically useful biomarkers or therapeutic targets in precision cancer research. The importance of the review is that it combines the existing evidence on the mechanistic and translational functions of macrophage-derived lncRNAs, and also reveals their potential as new controllers of tumor immunity and personal cancer therapy.

## 2 Macrophage-derived lncRNAs

This part of the review gives a background account of macrophage-derived lncRNAs and its fundamental regulatory characteristics before explaining their cancer-specific roles. Since these molecules can be transferred in EVs, and they can affect gene expression in many ways, they are significant mediators of macrophage-tumor communications. Knowledge of their overall biological characteristics is vital in making sense of their role in tumor progression and immune regulation in addition to therapeutic resistance in subsequent sections.

Their sizes differ in size and activities they regulate compared to shorter non-coding RNAs (ncRNAs), such as miRNAs (16). These molecules are involved in regulating chromatin, post-transcriptional regulation and transcription as either enhancers or repressors (17). Moreover, lncRNAs interact with RNA-binding proteins and change gene-specific, time-dependent epigenetic pathways, therefore acting as scaffolds (18). Among the macrophage-derived lncRNAs, NORAD, secreted by M2 macrophages, has been identified as essential for the advancement of NSCLC via transfer by EVs. Through interaction with SMIM22 and miR-520g-3p to encourage tumor development, it functions as a regulating element in processes like glycolysis, cell proliferation, and death (19). Driven in TNBC by M2 macrophage-secreted VEGF, lncRNA PCAT6 also stimulates angiogenesis, cell proliferation, and migration. PCAT6 reduces VEGFR2 expression and induces deubiquitination of VEGFR2 by way of a competitive endogenous RNA (ceRNA) mechanism and USP14 interaction, therefore increasing angiogenesis and tumorigenesis in TNBC (20, 21).

**Figure 1:**
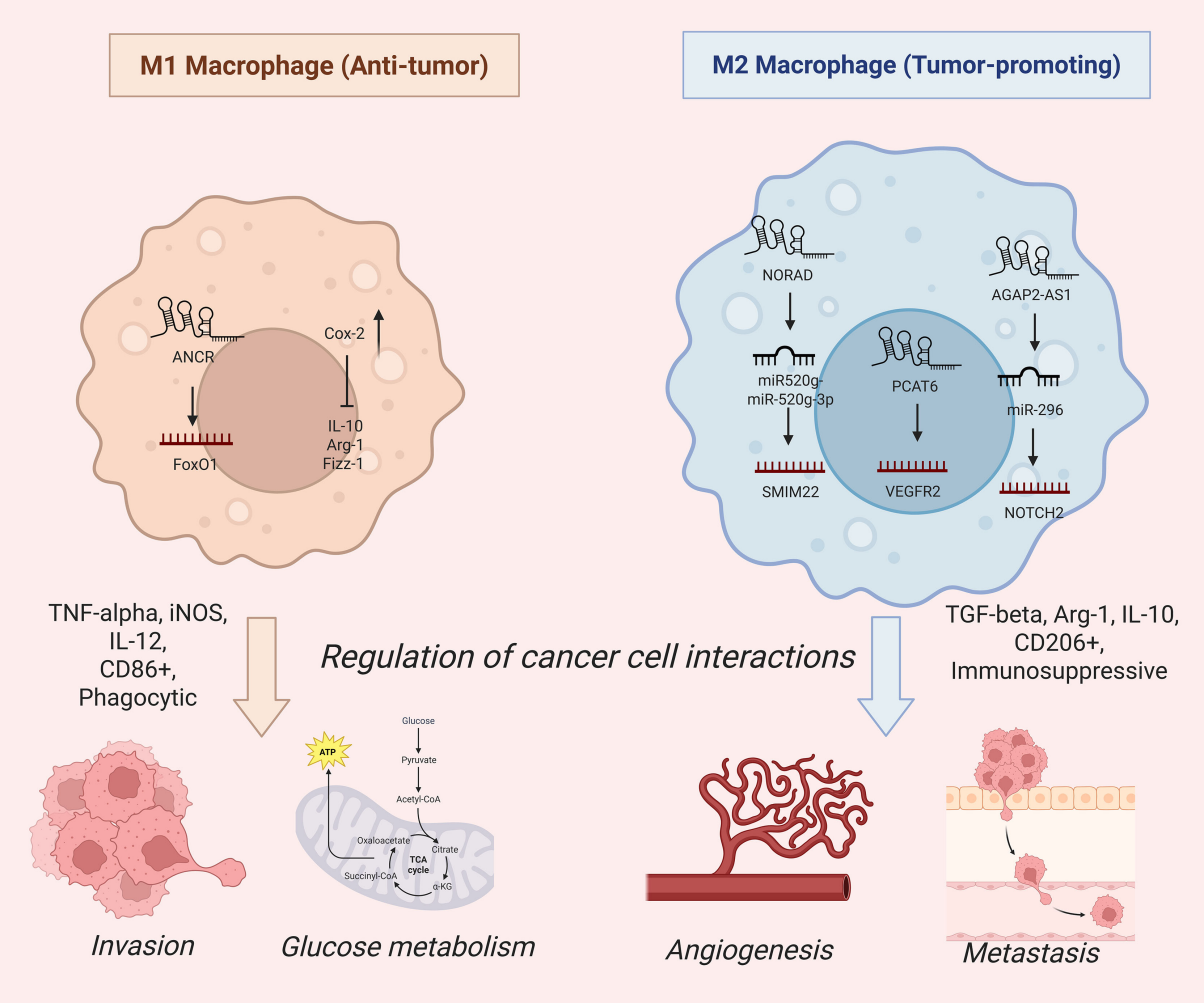
The diagram contrasts M1 (anti-tumor) and M2 (pro-tumor) macrophage polarization, highlighting key lncRNAs that drive their distinct effector profiles. M1 cells favors pro-inflammatory glycolysis with decreased TNF-α, IL-12, and iNOS levels, supporting phagocytosis and tumor cell killing. M2 cells favors oxidative metabolism, producing immunosuppressive mediators like Arg1, TGF-β, IL-10, and VEGF to promote angiogenesis, and metastasis.

Moreover, utilizing M2 macrophage-derived exosomes, lncRNA AGAP2-AS1 altered the miR-296/NOTCH2 axis to enhance radiation tolerance in lung cancer (LC). This link thus drives malignant behaviors of radioresistant LC cells and boosts immunity against radiotherapy (22). It has also been shown that Cox-2 displayed strong expression in M1 macrophages compared with M2, and its silencing leads to decreased expression of pro-inflammatory markers such as IL-12, iNOS, and TNF-α but promotes polarization toward the M2 phenotype with increased levels of IL-10, Arg-1, and Fizz-1 (23). Another notable macrophage-derived lncRNA is ANCR, which is upregulated in gastric cancer (GC) and has been recognized as a central factor in the regulation of macrophage polarization. It inhibits M1 polarization and reduces pro-inflammatory markers such as IL-1β and IL-6 while promoting tumor invasion and metastasis in GC by targeting FoxO1 for ubiquitination and degradation, thus suppressing its expression in macrophages (24). These discoveries have continued to emphasize the crucial functions of lncRNAs produced by macrophages in the immune regulation and the growth of tumors via complex regulatory connectors. Also, the macrophage polarization regulated by lncRNAs in the tumor microenvironment were depicted in **Figure 1**.

Altogether, an in-depth look at macrophage-derived lncRNAs demonstrates that they are multifunctional regulators that connect to tumor biology via transcriptional, post-transcriptional, and vesicle-mediated activities. This underlying position will be used to justify their new importance as mediators of macrophage plasticity and tumor-immune crosstalk. Based on this overview, the subsequent section explains the roles of specific macrophage-derived lncRNAs in cancer progression in M1-, M2-, and TAM-mediated processes.

## 3 Role of macrophage-derived lncRNAs in cancer progression

Macrophage-derived lncRNAs affect the progression of cancer in a polarization-dependent manner. These lncRNAs may facilitate anti-tumor inflammatory reactions or encourage immunosuppression, metastasis, and resistance to therapeutic agents, depending on whether they may be linked with M1 macrophages, M2 macrophages, or more general tumor associate macrophage populations. This part thus looks into their functions in key macrophage phenotypes to explain how macrophage plasticity triggered via lncRNAs impacts tumor behavior.

### 3.1 M1-derived lncRNAs

**Figure 2:**
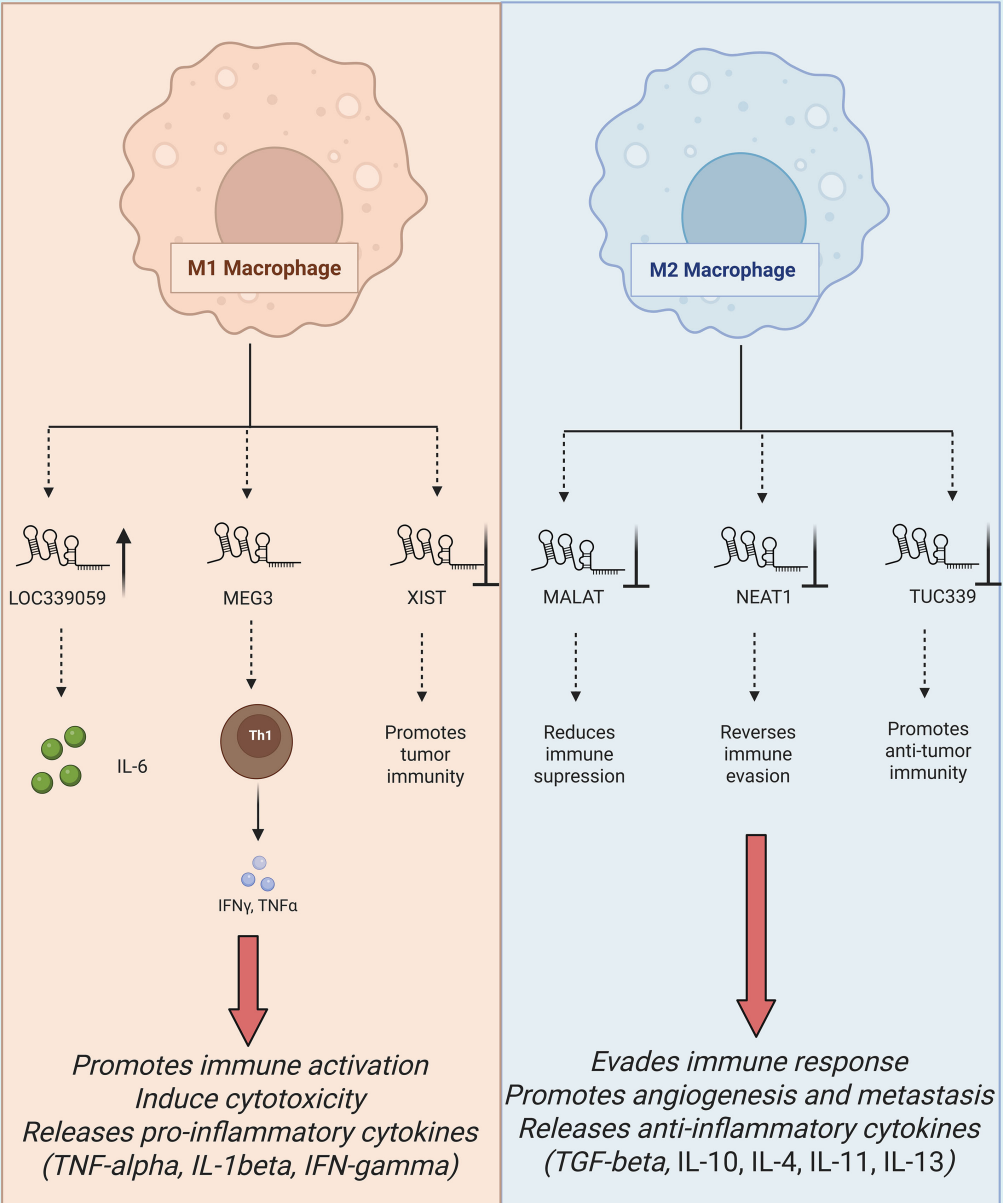
The diagram depicts the comprehensive ceRNA networks and regulatory mechanisms of macrophage-derived lncRNAs across M1, M2, and TAM phenotypes. Mainly M1 lncRNAs drive anti-tumor immunity, M2 lncRNAs promote tumor evasion and TAM lncRNAs sustains pro-tumorigenic TME crosstalk.

M1 macrophages are crucial in carcinogenesis in the sense that they induce a pro-inflammatory immune response that is also linked with inhibiting the progression of a tumor. These macrophages secrete such a huge number of cytokines and reactive oxygen species (ROS) as to destroy cancer cells (25). Several lncRNAs of M1 macrophages have also been found, and these are known to regulate this polarization and contribute to the anti-tumor activity. Inflammatory signaling also activates macrophages by such lncRNAs, making them play an important part in immune activity as immune modulators in cancer (26). **Figure 2** illustrates the M1-derived lncRNAs in cancer progression and TME regulation. Up-regulated lncRNA Nfkb2 is expressed in M1 macrophages due to the stimulation of a lipopolysaccharide (LPS). This lncRNA locates itself adjacent to significant transcription factors of the pro-inflammatory signatures and increases the degree of the inflammatory reaction via the regulation of the NF-ĸB signaling (27). TLR4 pathway results in up-regulation (enhanced synthesis) of transcription of pro-inflammatory forms of cytokines by the triggering of the NF-kB as aiding in combating tumors when induced by the M1 macrophages (28).

Similarly, M1 macrophages upregulate lncRNA Rel after activating TLR4, and this partnership is a collaboration with Nfkb2 to promote inflammation (29). All these lncRNAs are the elements promoting the synthesis of such cytokines as TNF-α and IL-1α, which, in turn, are the crucial factors that induce activation of the anti-tumor immune response, which attracts the immune cells and contributing to the death of the tumor cells (30). Another lncRNA of significance is AS-IL1α, which is a natural antisense transcript (NAT) and is involved in IL-1α, a pro-inflammatory cytokine regulation. AA-IL1 α induces AS-IL1 α to recruit RNA polymerase II (RNPII) in response to LPS stimulation to induce histone acetylation and IL-1α transcription at the IL-1α promoter (31). Overexpression is also helpful to establish the inflammatory conditions of the TME in the assistance of the anti-tumor activity of M1 macrophages. Further, lncRNA Cox-2 (also known as PACER) associates itself with the homodimeric complex of NF-κB, composed of p50 and p50, and activates the promoter of Cox-2, which is an inhibitor belonging to the NF-κB family (32). This aggravates the inflammatory process and contributes to the synthesis of the prostaglandins that facilitate the growth of the tumors. That PACER controls the expression of Cox-2 further lends credibility to the idea that it promotes inflammation among M1 macrophages and that it assists immune response to tumors (33).

The other lncRNA that was up-regulated in the LPS-treated M1 macrophage is called LincRNA-Tnfaip3. This lncRNA is involved in commencing inflammatory reactions by interacting with the high-mobility group box 1 (HMGB1), which allows the histone alterations and the attachment of the NF-kB subunits to DNA (34). The effect of this is more transcription of inflammatory cytokines, which are essential in the improvement of the macrophages’ anti-tumor immunity (30). Moreover, NF-κB in M1 macrophages has been found to bind to MALAT1 (Metastasis-Associated Lung Adenocarcinoma Transcript 1), which in turn prevents its binding to DNA, thus preventing the transcription of pro-inflammatory cytokines as well as growth factors such as TNF-α and IL-6 (29). This check makes sure that the inflammation process is maintained at low levels so that it does not develop severe inflammation that might cause damage to normal tissue (32). Finally, FIRRE (Functional Intergenic Repeat Element) binds to heterogeneous nuclear ribonucleoprotein U (hnRnPU), stabilizing inflammatory mRNAs and promoting a stronger inflammatory response of the M1 macrophage (35). It is this lncRNA that adds to the long-term presence of pro-inflammatory genes, which only increases the anti-tumor immune system supported by M1 macrophages (36).

### 3.2 M2-derived lncRNAs

M2 macrophages are readily related to tumor-enhancing events such as immune inhibition, remodeling of tissues, and augmentation of metastasis (**Figure 2**). The stimulus that usually activates macrophages to produce cytokines like IL-10 and TGF-β is mainly IL-4, IL-13, and immunological complexes that suppress immune response, thus leading to tumor propagation (37). Some lncRNAs of M2 macrophages serve as chief mediator of macrophage polarization and pro-tumor activities. These lncRNAs are important in the immunological TME as far as malignancy is concerned (38). Much like M1 macrophages, M2 macrophages can also be upregulated by Mirt2, and macrophage polarization and immunological responses are highly dependent on Mirt2. This lncRNA hampers the process of oligomerization and ubiquitination of vital protein in the NF-κB signaling cascade, which is TNF receptor-associated factor 6 (TRAF6) (39). Mirt2 aids in the alteration in the transition towards the more immunosuppressive phenotype by suppressing the primary inflammatory responses. Also, restoring Mirt2 protein expression in M2 macrophages enhances their polarization and the level of synthesis of key M2 markers, such as IL-10 and Arg1 (40). The results show that Mirt2 is crucial for the stability of the M2 phenotype, especially in inflammatory situations like malignancies, where suppression of the immune system promotes the growth of the tumor.

HOTTIP (HOX Transcript Antisense Intergenic RNA) was found to correlate with M2 polarization of macrophages, particularly in endotoxin tolerance, a phenomenon of reduced response of macrophages to LPS (41). HOTTIP is revealed to control immunological tolerance in endotoxin-tolerant macrophages by switching macrophages to an M2-like phenotype. The ability of HOTTIP to alter macrophage responses provides evidence of the HOTTIP activity as an important regulator in chronic inflammation and TME, where the continued immune suppression effect would be conducive to the fostering of tumor growth and immune evasion (42). KCNQ1OT1 is an interesting lncRNA that has been discovered to mediate M2 macrophage polarization. KCNQ1OT1 has also been described as a miRNA miR-21a-5p decoy in studies of polymethyl methacrylate (PMMA)- induced bone marrow-derived macrophages (BMDMs) (43). KCNQ1OT1 sequesters miR-21a-5p, the sequestration of which indirectly increases IL-10 and induces the M2-macrophages (44). The approach suggests that KCNQ1OT1 promotes M2 polarization through enhanced anti-inflammatory signaling as a vital immune evasion mechanism in cancer. The elevation of IL-10 as a result of KCNQ1OT1 also confirms the immunosuppressive role of M2 macrophages within the TME (45). Intermediate TCONS_00019715 controls the M/M2 macrophage switch. There is an up-regulation of this lncRNA during a transition of M2 to an M1 phenotype, but its expression decreases once M1 macrophages renounce their M1 phenotype (46). This dynamic regulation implies that TCONS_00019715 needs macrophage polarization to be sustained throughout a tumor-related response to tumor-associated cues (47). The expression of the TCONS 00019715 may provide a clue to the recruitment and retention of macrophage plasticity in the TME that may impact tumor expansion and immune reaction (48).

MM2P lncRNA is an important regulator of the polarization of M2 macrophages. M2 polarization increases MM2P and contributes to the phosphorylation of a transcription factor, STAT6, which is unequivocally important in the activation of M2 macrophages (49). M2 polarization occurred less in the RAW264.7 cells where MM2P was silent, and this is evidenced by the lowering of M2 markers, including IL-10 and Arg1 (50). The effect of evidence indicates that MM2P is very instrumental in upholding M2 phenotype with reference to the signaling pathways MM2P plays with regard to the immune suppression and tissue repair processes of these malignancies (51). GAS5 is a lncRNA, which has been demonstrated to alter macrophage polarization in regard to cancer and inflammation (52). In pneumonia patients, GAS5 is overexpressed in monocyte-derived macrophages as miR-455-5p decoys (53). GAS5 increases SOCS3 expression by sponging miR-455-5p, thereby blocking the JAK2/STAT3 pathway, a process that promotes M2 polarization. By such a regulation, GAS5 shapes the polarization of macrophages and helps to suppress the immune response to cancer, therefore allowing the tumors to grow and metastasize (54). This explains how GAS5 is relevant in terms of immune escape of malignancies. The lncRNAs of M2 origin included Mirt2, HOTTIP, KCNQ1OT1, TCONS_00019715, MM2P, and GAS5, and are important in regulating a shift by macrophages to the M2 phenotype, which is characterized by a state of immunosuppression and progression of cancer (55). More recent syntheses devoted to breast cancer also confirm the idea that the macrophage polarization under the influence of lncRNAs is extremely context-dependent and clinically significant. Ghorbaninezhad et al. emphasized the ability of breast cancer-related lncRNAs to shift macrophages into tumor-friendly phenotypes to support invasion, metastasis, angiogenesis, and resistance to therapy (56). Simultaneously, Patni et al. highlighted that ncRNA-mediated TAM plasticity in breast cancer is closely connected with the exosomal signaling and can be clinically utilized by means of targeted nanodelivery systems aimed at reprogramming the M2-like macrophages located in the TME (57). Notably, recently discovered therapeutic research have shown that inverting polarization into M2 or M1 reprogramming may go a long way in boosting anti-tumor immune response as well as the therapeutic response, and that plasticity of the macrophages seems to be a therapeutic target in the clinical setting (58, 59).

We believe that overall, the above reviewed studies suggest that lncRNAs can be seen as upstream regulators of macrophage polarization and not passive downstream responses of M2 activation. One common trend emerging in these reports is that lncRNAs strengthen immunosuppressive macrophage identity by converging on a small number of regulatory circuits, such as the attenuation of NF-kB, the activation of STAT3/STAT6, remodeling of cytokines and deregulation of pro-tumor genes by ceRNAs. This implies that it is not a single pathway that drives the process of macrophage polarization in cancer, but rather multilayered lncRNA-centered networks that trap macrophages into tumor-supportive modes. Our belief is thus, that future studies ought to focus on revealing the identities of lncRNAs that are functionally necessary to support macrophage plasticity, since these might be more therapeutically relevant than those lncRNAs which simply vary with polarization.

### 3.3 TAM-derived lncRNAs

TAMs comprise a significant component of the TME and influence cancer formation, immune evasive mechanisms, metastasis, and drug resistance to a large extent (60). TME exerts its regulatory effect on macrophages that have the potential to become either pro-inflammatory M1 or immunosuppressive M2 (61). Several factors influence polarization of TAMs, and among them, lncRNAs are gaining significance in tumor immunology (62). TAM-derived lncRNAs have a direct effect on macrophage polarization, the ability to restructure the immunosuppressive environment of the TME, and support tumor progression by transcriptional and post-transcriptional regulation of gene expression (63). One of the most described lncRNA-derived in TAM is HISLA, which was differentiated in breast cancer. The TAMs use EVs, or herein referred to as exosomes, in order to get HISLA empty, as it stabilizes the transcription factor known as hypoxia inducible factor 1-alpha (HIF-1α), which contributes to the acquisition of hypoxic adaptation (64). HISLA improves the survival of tumor cells and promotes aerobic glycolysis by preventing the deposition of HIF-1α. This lncRNA promotes the positive feedback loop (65). The secreted lactate by tumor cells induces the production of HISLA by TAMs and thus produces a favorable tumor-promoting environment (66). HISLA is linked to an unfavorable prognosis in breast cancer, and its role in chemotherapy resistance suggests that HISLA is a viable target of therapeutic interventions (67). The more recent literature on breast cancer suggests that the TAM-lncRNA environment is not limited to HISLA. Recent surveys report other breast cancer-linked lncRNAs, such as LINC00337, LINC00657, GNAS-AS1, lncRNA-109, and LINC00514 as regulators of macrophage polarization, cytokine restructuring and chemoresistance (57). All of these results outline a picture according to which in breast cancer, TAM-derived or TAM-modulating lncRNAs do not exist as independent entities, but these represent the parts of larger communication cascades involving exosomes, macrophage states change, and tumor-cell fitness. Another one that is upregulated in the TAMs of pancreatic cancer is the SBF2-AS1 (68). The lncRNA acts as a ceRNA, binding to the miRNA, miR-122-5p, to sequester it, an anti-apoptotic protein-binding miRNA, such as XIAP (69). SBF2-AS1 indirectly elevates the expression level of XIAP via blockade of miR-122-5p, which helps tumor cells to survive and be resistant to chemical drugs (70). By therapeutically blocking the SBF2-AS1 in TAMs, the tumor-inhibitor role of miR-122-5p is maximized, hence, reducing the development of pancreatic cancer, and therefore demonstrating the possibility of using this lncRNA in therapy (71).

MALAT1 is secreted in an M2 phenotype of TAMs in GC. MALAT1 leads to a significant realization of inflammation by induction of the NF-κB signaling pathway (72). The lncRNA increases the synthesis of the TNF-α and IL-6, leading to the survival of the tumor cells, metastasizing, and showing resistance to chemotherapy (73). It has been suggested that the presence of MALAT1 in the stomach and LCs has been a determinant of chemoresistance to drugs such as 5-FU and cisplatin, which makes it the key determinant of treatment failure. The fact that MALAT1 is relevant in inflammatory responses in the TME means that it can be used in overcoming chemoresistance (74). H19 is an lncRNA that is up-regulated by TAM and was implicated in mediating both instances of metastasis and chemoresistance in colorectal and breast cancers. H19 activates the β-catenin signaling pathway that regulates cell adhesion, cell migration, and cell invasion activities (75). H19 enhances the invasion of self-absorbing cells and metastatic dissemination of tumorous cells by modulating β-catenin. Moreover, H19 has been demonstrated to promote chemotherapy resistance, thus becoming the determinant of tumor-supportive function of TAMs (76). The importance of H19 to regulate cell attachment and migration means that its targeting can prevent the metastaticity of malignancies (77).

Furthermore, M2-polarized TAMs exosomes are rich in CRNDE (Colorectal Neoplasia Different from Expression). Pathologically, CRNDE contributed to enhance cisplatin insensitivity in GC through the role-playing of PTEN/NEDD4–1 signal factors, which mediate the survival/death processes of tumor cells (78). CRNDE also increases tumor cell survival mediated by TAMs, as it promotes chemotherapy resistance, indicating the importance of TAMs in the advancement of therapy insensitivity in the TME (79). AGAP2-AS1 is a lncRNA that has been shown to regulate the miR-296/NOTCH2 axis and alter tumor development and radiotherapy tolerance in radioresistant LC cells (80). AGAP2-AS1 contributes to the modulation of the TME by enhancing radiation resistance, and, thus, the clinical promise of lncRNA targeting in TAMs to overcome anti-cancer resistance paradigms (81). Finally, a considerable lncRNA that interferes with TAM behavior within the TME is HOTAIR (HOX Transcript Antisense Intergenic RNA). HOTAIR intensifies the invasion of the TAMs and increases the proportion of the myeloid-derived suppressor cells (MDSCs) that provide immune suppression in malignancies (82). HOTAIR promotes immune evasion and metastasizes, therefore promoting the pro-tumorigenic activity of TAMs and facilitating cancer growth and spread. Furthermore, pharmacological therapy that limit the infiltration of macrophages or causes changes in the macrophage-mediated metabolic processes have been demonstrated to inhibit tumor progression, adding more support to the pivotal role of macrophages in tumor biology (83). It is also able to control the polarization process of macrophages and immunological response, making it an important target of methods aimed at reprogramming TAMs in cancer therapy (84). Besides the lncRNA-mediated signal, M2 macrophage-secreted EVs have even been reported to have a direct promoting angiogenesis effect, via HIF-1α/VEGFA signaling, which further highlights the pro-tumorigenic nature of macrophage-secreted EVs (85). In addition to the studies described above, **Table 1** presents additional macrophage-derived lncRNAs along with their mechanisms and roles in cancer progression.

**Table 1:** Macrophage-derived lncRNAs, their mechanisms, and roles in cancer progression.

lncRNA	Cancer/type	Macrophage type	Mechanism	Role	Reference
lncMMPA	Hepatocellular carcinoma	M2-polarized TAMs	Exosomal delivery; functions as a competing endogenous RNA (ceRNA) for miR-548s, leading to ALDH1A3 upregulation	Enhances glycolysis, proliferation, and M2 polarization	(86)
NEAT1	Hepatocellular carcinoma	M2-polarized TAMs	Exosomal transfer; recruits KLF5 to the LGALS3 promoter, resulting in increased galectin-3 expression	Promotes proliferation, migration, and immune evasion	(87)
NEAT1	Inflammatory bowel disease–related colorectal cancer	TAMs from IBD-CRC tissues	Exosomal transfer; acts as a sponge for miR-34a-5p, restoring PEA15 expression	Enhances cancer stemness and tumor growth	(88)
CRNDE	Glioma	M2-polarized TAMs	Promotes M2 macrophage polarization and modulates the tumor microenvironment	Facilitates proliferation, invasion, and malignant progression	(89)
LINC01569	Hypopharyngeal carcinoma	M2-polarized TAMs	Acts as a sponge for miR-193a-5p, leading to upregulation of FADS1	Promotes tumor growth and immune escape	(90)
MIR4435-2HG	Infantile hemangioma	M2-polarized TAMs	Exosomal delivery; binds to HNRNPA1 and activates the NF-κB signaling pathway.	Promotes proliferation, migration, invasion, and angiogenesis	(91)
LRRC75A-AS1	Cervical cancer	M2-polarized TAMs	Exosomal transfer; suppresses miR-429 expression, resulting in increased SIX1 levels and activation of the STAT3/MMP-9 axis	Facilitates proliferation, epithelial-mesenchymal transition (EMT), and metastasis.	(92)
NR_109	Gastric and breast cancer	M2-polarized TAMs	Stabilizes FUBP1 by competing with JVT-1, preventing its ubiquitin-mediated degradation, and activates c-Myc transcription	Promotes proliferation, metastasis, and M2 polarization	(93)
LINC01001	Non-small cell lung cancer	M2-polarized TAMs	Exosomal delivery: interacts with METTL3 and regulates NASP methylation, enhances glycolysis, and inhibits T cell function	Promotes glycolysis, proliferation, and immune suppression	(94)
SNHG17	Pancreatic ductal adenocarcinoma	M2-polarized TAMs	Acts as a sponge for miR-628-5p, leading to increased PGK1 expression and enhanced phosphorylation via ERK1/2 signaling	Promotes M2 polarization, glycolysis, and metastasis	(95)
SNHG1	Breast cancer	M2-polarized TAMs	Enhances STAT6 phosphorylation, promoting M2 macrophage polarization	Supports tumor growth, metastasis, and angiogenesis	(96)
MEG3	Hepatocellular carcinoma	Bone marrow-derived macrophages	Binds to HuR, inhibits CCL5 transcription, shifts macrophages toward M1 polarization	Suppresses migration, invasion, and tumor growth	(97)
MEG3	Hepatocellular carcinoma	THP-1 macrophages	Reduces CSF-1 and PD-1/PD-Ls expression, enhances M1 markers and Th1 cytokines, suppresses M2-like polarization	Inhibits invasion, tumor growth, and promotes anti-tumor immunity	(98)
Myt1l	Pancreatic cancer	M2-polarized TAMs	Exosomal transfer; acts as a ceRNA for miR-135, resulting in upregulation of Slug and increased vimentin expression	Promotes migration, invasion, and metastasis	(99)
LINC01232	Glioma	TAM-derived exosomes	Binds to E2F2, facilitates its nuclear translocation, and promotes NBR1 transcription, leading to MHC-I degradation	Promotes immune evasion and impairs CD8+ T cell recognition	(100)

To our minds, the evidence examined in the context of M1-, M2-, and TAM-derived lncRNAs promotes a unifying idea; lncRNAs act as regulation nodes that assist in preserving the macrophage phenotypic plasticity within the TME. Even though most investigations characterize individual lncRNAs individually, the overall trend involves a focus on a small set of biological outputs, such as the strengthening of M2-like immunosuppressive restorations, the dampening of inflammatory anti-tumor signaling, the amplification of tumor-cell survival mechanisms, and the boosting of treatment resistance. We thus feel that this field should not be viewed informatively as a growing list of single-isolated lncRNAs, but a network of context-sensitive regulators that set macrophages in tumor-supportive programs. This view also underscores the reason why macrophage-derived lncRNAs can be of particular significance in malignancies in which immune evasion and stromal interactions are key triggers of the disease.

Collectively, the facts considered in this section confirm that the macrophage-derived lncRNAs are the decisive factors in cancer development since they dictate the acquisition of tumor-suppressive or tumor-promoting capabilities of macrophages. One of the central ones is that such lncRNAs drive context-dependent immunomodulatory activities, macrophage differentiation, metabolic reconfiguration, and resistance-associated pathways. It is this mechanistic diversity that gives them their wider implications on immune-regulation that are described in the following section.

## 4 Effect of macrophage-derived lncRNAs on the immune system

In addition to their direct implications on tumor growth, macrophage-derived lncRNAs have extensive implications on immune regulation in the TME. These lncRNAs regulate both innate and adaptive immune responses by regulating macrophage polarization, cytokine secretion, chemokine signaling, immune checkpoints, and epigenetic programs. This segment briefly describes the role of macrophage-derived lncRNAs in regulating immune behavior and causing chronic immune dysfunctions in cancer.

Investigating immune dysregulation and its relevance in many clinical states, including inflammation, cancer, and autoimmune diseases, depends on an expanding field of research on how lncRNAs originating from macrophages affect the immune system (101). Natural immune system components are macrophages, which are essential. They control tissue homeostasis and immunological responses. Recent research has explained various methods of regulating the immunological responses (102).

Macrophage-derived lncRNAs are essential to control macrophage polarization towards specific phenotypes. These phenotypes, each performing distinctly different roles in tissue healing, inflammation, and immunological control, are M2 activated alternatively, and M1 activated traditionally. As an example, M1 polarization through specific lncRNAs, e.g., MEG3 and GAS5, reduces negative regulators of M1 genes or Wnt/-catenin signaling pathway deactivation, which has been recognized to induce tumor cell apoptosis (53, 103). On the other hand, the presence of lncRNAs like MALAT1 and TUG1 characterizes macrophage differentiation into the M2 type related to tumor creation, tissue remodeling, and immunosuppression. The method amplifies the production of immunosuppressive TME M2 macrophages through the initiation of discrete signaling pathways or lncRNAs that act as ceRNAs. This highlights the several complicated ways in which lncRNAs generated by macrophages alter the TME and regulate immune reactions (104, 105). In line with this, M2-macrophage extracellular vesicles have been identified to control the activity of other immune cell groups and, as such, the macrophage-produced factors that mediate systemic immunoregulation (106).

We interpret it that the immunological role of macrophage-derived lncRNAs can be seen most usefully in terms of their power to mediate polarization state to downstream immune action. That is, the lncRNAs are not just the determinant of the existence of a macrophage as being M1 and M2-like: they also dictate the existence of the phenotype under the tumor-induced pressure and how well it can modify the activity of T-cells, cytokine homeostasis, and immune checkpoint signaling. It is biologically significant that TAMs exist in intermediate and transitional states *in vivo*, and lncRNAs possibly give the regulatory subsistence required to support these phenotypes. In this way, the regulation of macrophage polarization through the lncRNAs can be one of the key questions of how the TME maintains a state of chronic immune dysfunction.

Especially controlling the production of cytokines and chemokines, basic modulators of inflammation and immune cell interaction. The interaction between cancer development and inflammatory process is further proved by the research that shows that bioactive molecules (heparin derivatives) may regulate the inflammatory signaling cascades and may modify tumor development (107). LncRNAs control the recruitment, activation, and activity of several immune cell types, such as macrophages, T-cells, B-cells, and dendritic cells, employing their regulatory mechanism. Diverse events associated with specific lncRNAs have diverse effects: autoimmune reactions, chronic inflammation, or improved antiviral responses. In viral infections, lncRNA-Neat1, for instance, boosts the pro-inflammatory cytokine IL-1β, therefore enhancing the immune response and enabling virus elimination (108). Conversely, lncRNA-Chaer1, greatly raised in pathogenic diseases like atherosclerosis, promotes the synthesis of the chemokine CXCL12, which in turn helps monocytes to be recruited and accelerates the development of chronic inflammation and plaque formation (109). Furthermore, lncRNA-MEG3 functions as an anti-inflammatory regulator in autoimmune illnesses by reducing macrophage synthesis of pro-inflammatory cytokines and so minimizing tissue damage caused by too strong inflammation (110). These explanations show the complicated role lncRNAs produced from macrophages, are vital in influencing the immune response; based on the particular lncRNA and the surroundings in which it operates, it may either aggravate or reduce pathological inflammation.

Macrophage lncRNAs control immune cell migration and entrance into inflammatory microenvironments or contain malignancies. LncRNAs generated from macrophages influence the quantity and structural arrangement of immune cell populations within tissues using direct interaction with other immune cells or manipulation of chemokine signaling pathways, therefore modulating the immunological activity. For instance, lncRNA-MALAT1 can raise CCL2’s expression, a chemokine identified for attracting monocytes. Through subsequent differentiation into M2 macrophages within the TME, the invading monocytes help the tumor to grow by utilizing immunosuppressive processes (111).

LncRNAs produced from macrophages have recently been linked to the control of immunological checkpoint molecules. Maintaining immunological tolerance and preventing too strong an emphasis on immune activation depends on these molecules (36). Dysregulation of many checkpoints and immune evasion in cancer and autoimmune disorders are correlated. Therefore, lncRNAs obtained from macrophages can affect the functioning and control of immunological checkpoints, possibly affecting the efficiency of immunotherapy approaches. More and recent prognostic and immunotherapeutic evidence has shown immune-related lncRNA signatures have prognostic and immunotherapeutic value across cancers due to their recent computational and transcriptomic applications (112). Previous research showed that reducing the expression of lncRNA XIST increases the synthesis of tumor-secreted exosomal miR-503, hence regulating PD-L1 on M2 macrophages and preventing T-cell proliferation in BC (113).

Utilizing many epigenetic mechanisms, including chromatin remodeling, histone modification, and DNA methylation, macrophages produce LncRNAs that alter immune cell gene control. Changes in the cellular landscape form influence several components of the immune system since they influence the way cells decide their fate as well as their differentiation routes and functional results. The action of the well-known MALAT1 causes the anti-inflammatory cytokine gene IL-10 produced in immune cells to show increased histone acetylation control. Through this technique, chromatin modification allows transcription factors to reach genes, thereby enabling access to initiate processes of expression (114). Notably, we would consider that the immunological effect played by the macrophages-derived lncRNA is not limited to positive control of cytokine expression. These molecules seem to offer mechanistic connectivity between the macrophage polarization condition and tumor microenvironment downstream immune design as T-cell stifling, chemokine-mediated leukocyte recruitment, checkpoint, and inflammatory retention. This could be the reason why the lncRNAs dysregulation in general is accompanied by the disrupted macrophage identity and by the general immune dysfunction. We believe that the biological importance of lncRNAs is that they can maintain maladaptive immune responses over time, and therefore can be considered to be more than simple indicators of macrophage activation. Ultimately, in immune cell biology, lncRNAs from macrophages create a complicated and fascinating regulatory layer. Knowing the roles and imbalance of these lncRNAs in immune cells can provide important new perspectives on the causes of immunologically related diseases and guide the creation of creative treatment strategies aiming at immune system modification.

Together, the above studies suggest that macrophage-derived lncRNAs are not independent mediators, but collectively integrate into few physical axes of immunoregulation: macrophage polarization, cytokine and chemokines production, immune checkpoint regulation, and inflammatory programs epigenetic regulations. There is also a mechanistic similarity, that most lncRNAs play a role by ceRNAs or chromatin-bound regulators that stabilize pre-existing tumor-imposed macrophage states already. This implies that lncRNAs do not simply reflect the activation of TAM, but they serve as active stabilizers of pro-tumor or anti-tumor immune phenotypes. Simultaneously, the analysis of the literature should be performed with care since the biological activity of a particular lncRNA can be very context-specific, as different types of tumors, different stages, and sources of macrophages, and different experimental platforms may yield different outcomes. As an example, the lncRNAs MALAT1, GAS5, or XIST have been associated with immune regulation in various disease environments, but their downstream effects are not necessarily the same, implying that the lineage context and microenvironmental clues potently influence functions. Thus, the field must shift past lncRNA cataloguing and to specifying which lncRNAs are causal regulators of the plasticity of macrophages, which are secondary readouts of the activation state and which are strong enough to be therapeutic actionable nodes in tumor immunity.

## 5 Therapeutic potential of targeting macrophage-derived lncRNAs

Given their central roles in macrophage polarization, tumor-immune communication, and therapy resistance, macrophage-derived lncRNAs have emerged as potential therapeutic targets in cancer. The reasoning behind the use of these molecules is that the modulation of a subset of lncRNAs can re-program TAMs, re-establish anti-tumor immunity, and enhance sensitivity to the available treatments. The novel treatment modalities, such as the use of oncolytic peptides and engineered biologics, also underscore the significance of targeting the tumor-immune interactions in order to increase the anti-cancer effects (115). This section highlights representative therapeutic studies and considers the translational significance of lncRNA-directed strategies.

Targeting macrophage-derived lncRNAs is an emerging treatment approach for cancer. The most important point about it, however, is that therapeutic relevance does not depend only on whether a lncRNA can modify tumor behavior, it also depends on whether it takes up a non-redundant regulatory role in macrophage biology. Numerous lncRNAs have been reported to regulate tumor progression via ceRNA interactions, modulation of NF-kB, STAT3/STAT6, HIF-1α or via checkpoint-related signatures; however, these same pathways are regulated by multiple upstream inputs, suggesting the chance of a compensatory response to the loss of a specific lncRNA. Hence, therapeutic candidates that meet three criteria are likely to be the most promising: the macrophage-enriched expression, the reproducible mechanistic connection between the tumor-promoting expression of TAM phenotype and the ability to reverse immune suppression *in vivo*.

**Figure 3:**
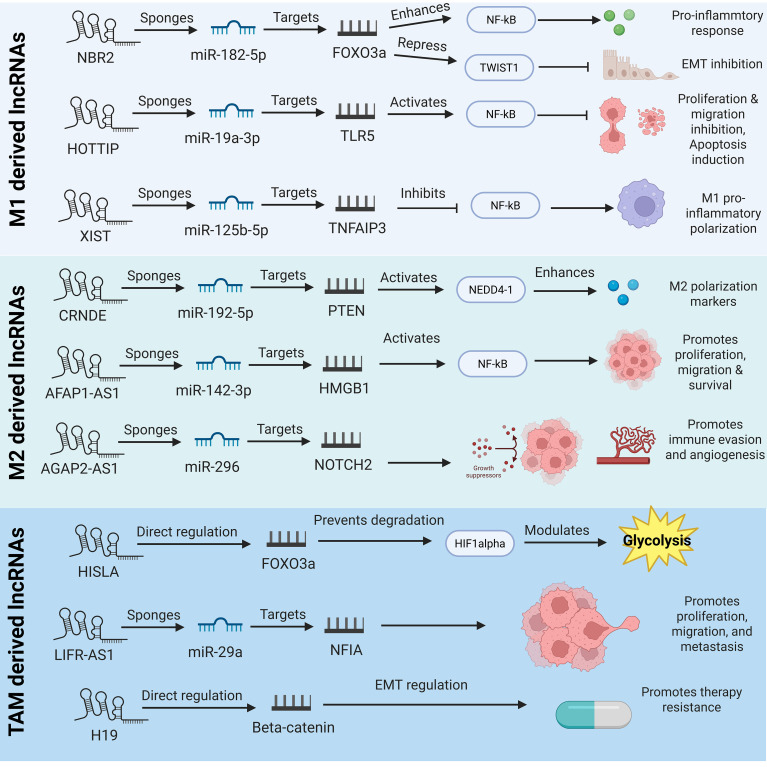
The diagram highlights the opposing roles of polarization-specific lncRNAs from M1, M2, and TAMs in tumor progression and TME modulation. M1-derived lncRNAs drive cytotoxic cytokine release (TNF-α, IL-6) to suppress metastasis and growth. M2-derived lncRNAs promote immune evasion and angiogenesis. TAM-derived lncRNAs reinforce immunosuppression, highlighting therapeutic reprogramming opportunities.

As illustrated in **Figure 3**, some specific lncRNAs, including TUC339, MEG3, MALAT1, NEAT1, XIST, and LOC339059, can modulate macrophage polarization and tumor immune responses. Recent research has demonstrated that targeting specific lncRNAs in macrophages can efficiently inhibit tumor growth, overcome drug resistance, and stimulate antitumor immunity. For example, inhibition of M1 macrophage-exosomal lncRNA HOTTIP exhibited strong antitumor efficacy in HNSCC by suppressing tumor cell replication and migration and activating TLR5/NF-κB signaling pathway via sponging miR-19a-3p and miR-19b-3p (28). Conversely, knocking down M2 macrophage-exosomal lncRNA SBF2-AS1 significantly inhibited pancreatic cancer development by blocking its role as a ceRNA for miR-122-5p, suppressing the levels of XIAP, and preventing cancer cell survival (116). Similarly, therapeutic suppression of tumor-related macrophage-derived exosomal lncRNA LIFR-AS1 suppressed osteosarcoma cell development and metastasis by disrupting the miR-29a/NFIA pathway (26). Moreover, blocking M2 macrophage-derived EV lncRNA NORAD in NSCLC suppressed tumor glycolysis and growth by restoring the activity of miR-520g-3p and suppressing SMIM22 and GALE expression (19). Additional studies involving various cancer types and underlying mechanisms are comprehensively summarized in **Table 2**, providing an extended overview of macrophage-derived lncRNAs as actionable therapeutic targets. This therapeutic view is also coherent with the wider perspective that intercellular communication by extracellular RNA can promote active influence of treatment response in cancer. In our last study, we have explained the role of extracellular RNAs in chemotherapy resistance involving dynamic communication between cancer cells and the nearby microenvironment (1). We feel that macrophage-derived lncRNAs are a better representation of this principle wherein non-coding RNA exchange by macrophages can fortify tumor survival, immune evasion, and resistance-reliant phenotypes. This further context sustains the belief that not only are macrophage-derived lncRNAs mechanistically relevant, but in addition, they are functional therapeutic parts of the resistant tumor ecosystem.

**Table 2:** Overview of macrophage-derived lncRNAs as therapeutic targets in cancer.

lncRNA	Cancer type	Macrophage subtype	Mechanism	Reference
HISLA	Bladder cancer	TAMs	Stabilizes β-catenin, promotes EMT and metastasis	(117)
MEG3	Prostate cancer	M1	Sponges miR-148a-3p, upregulates ARRDC3, promotes M1 polarization, inhibits M2 polarization and tumor progression	(118)
XIST	Lung cancer	M2	Transcriptionally regulated by TCF-4, promotes M2 polarization, enhances tumor progression	(119)
MEG3	Hepatocellular carcinoma	M2	Binds miR-145-5p, upregulates DAB2, suppresses M2 polarization, inhibits tumor growth	(120)
AGAP2-AS1	Lung cancer (radioresistance)	M2	Sponges miR-296, upregulates NOTCH2, promotes radioresistance	(22)
AFAP1-AS1	Esophageal cancer	M2	Sponges miR-26a, upregulates ATF2, promotes migration and metastasis	(121)
CRNDE	Gastric cancer (cisplatin resistance)	M2	Promotes NEDD4-1-mediated PTEN ubiquitination, enhances chemoresistance	(122)
Lnc-Meg8	Salivary adenoid cystic carcinoma	TAMs	Sponges miR-148a-3p, upregulates EGFR, promotes EMT, and metastasis	(123)
CRNDE	Liver cancer	M2	Promotes M2 polarization and angiogenesis	(124)
HOXC-AS2	Non-small cell lung cancer	TAMs	Binds STAT1, promotes M2 polarization, enhances tumor progression	(125)
MALAT1	Gastric cancer	M2	Sponges miR-217-5p, upregulates HIF-1α and β-catenin, promotes glycolysis	(25)
MEG3	Hepatocellular carcinoma	M1	Inhibits CSF-1, promotes M1 polarization, suppresses tumor growth	(98)
ADPGK-AS1	Lung cancer	M2, TAMs	Binds MRPL35, enhances TCA cycle & fission, promotes M2 polarization and tumor progression; silencing reprograms to M1 phenotype	(126)
MALAT1	Thyroid cancer	TAMs	Promotes FGF2 secretion, shifts cytokine profile, enhances tumor progression	(127)

Despite these encouraging results using macrophage-derived lncRNAs as therapeutic targets, there are a number of real-world challenges that bind the translation. First, TAMs are heterogeneous and dynamically reprogrammed, meaning that a target identified in one macrophage subset or cancer type may not be uniformly relevant across tumors. Second, delivery is one more significant issue, lncRNA-targeted procedures have not only to enter the TME efficiently but also to have no off-target action in the rest of immune or stromal cells. Third, since a significant number of lncRNAs are engaged in more global homeostatic and inflammatory interactions, an overall inhibition would have unintended immunological effects. Lastly, a great deal of the evidence relies on cell lines, xenograft models or exosome-transfer systems which might not be able to fully recapitulate the complexity of human TAM. Therefore, the translational relevance of this area is not necessarily the identification of additional lncRNAs, but rather the prioritization of mechanistically consistent, cell-type specific, therapeutically tractable lncRNAs in clinically-relevant models.

Regarding therapy, we consider that the most useful macrophage-derived lncRNAs do not necessarily represent the ones with the greatest expression variation, but the ones that are allowing central regulatory roles in polarization regulation. We believe that lncRNAs that concomitantly regulate macrophage identity, exosomal communication, and resistance-associated pathways will be found to have the highest translational utility. These types of molecules could facilitate reprogramming of TAMs non-persistent M2 phenotypes to anti-tumor immune response. This will however necessitate close differentiation between lncRNAs that may be causal in terms of macrophage polarization, and those that may in turn be secondary manifestations of tumor-induced activation states. Altogether, recent results indicate that macrophage-derived lncRNAs are prospective, yet unexploited, therapeutic targets. They have potential because they can be used to regulate macrophage phenotype, disrupt tumor-promoting signaling as well as overcome therapy resistance but they will require better target validation, superior delivery schemes and cell-type selectivity before clinical exploitation. These translational considerations do indeed spill over into the topic of personalized medicine in the next section.

## 6 Personalized medicine and lncRNAs

There is increased interest in macrophage-derived lncRNAs in more than just the mechanism but also precision oncology. Since they are capable of demonstrating macrophage state, tumor immunologic context and treatment sensitivity, it may become future biomarkers of prognosis, that can be used to stratify patients and as biomarkers to make treatment decisions. This section talks about the possibility of using macrophage-derived lncRNAs to help develop more personalized ways of cancer diagnosis and treatment.

Emerging as crucial controllers of cancer progression, macrophage-derived lncRNAs provide exciting paths for immune modulation and tailored therapy options. Yet, this kind of personalized medicine concept needs to be stratified better. The macrophage-derived lncRNAs may become at least three separate clinical applications, prognostic biomarkers of TAM burden or immune suppression, predictive biomarkers of patients more likely to respond to immunotherapy or macrophage-reprogramming approaches, and therapeutic targets themselves. The difference between these roles is necessary, as not all lncRNAs that correlate with poor outcome are automatically susceptible to intervention, and not all mechanistically relevant lncRNAs will be strong enough to be used as clinical biomarkers.

In one study, hepatocellular carcinoma (HCC) showed that ELMO1-AS1, which is a macrophage-related lncRNA, was particularly downregulated. Its higher expression was associated with better patient outcomes, which showed that ELMO1-AS1 could be a predictive biomarker. Suggesting its potential therapy, it is overexpressed in cells that facilitate the growth, invasion, and metastasis of HCC (128). The macrophagic LINC01592 lncRNA also played a role in immune evasion in esophageal cancer. M2 TAMs delivered exosomes to tumor cells, which augmented NBR1 knowledge and reduced MHC-I, as such, promoted immune evasion in tumors. The axis LINC01592/E2F6/NBR1/MHC-I is one such target proposed as a customized cancer treatment (129). Moving on to the study with BC, the lncRNA Individualization (LncRInd) technique revealed subtype-specific lncRNAs, thus informing about personalized therapy decisions. The mesenchymal subtype that was observed in TNBC, which is rich in M2 macrophages, exhibited aggressive tumor behavior through PTOV1-AS1.

In comparison, the immunological subset associated with the CD4+ T cells was observed to be genetically unstable, and immune checkpoints were also activated, indicating that immunotherapy may be an applicable approach. Moreover associated with the resistance to paclitaxel is PRRT3-AS1, which further reinforces the forecasting ability of lncRNAs in guiding personalized therapies (130). Further studies of the modulatory roles of MALAT1 and HOTAIR in BC have discovered the incorporation of macrophages in tumor growth. Significant alterations to central immunomodulating proteins, such as CD80 and mesothelin, in TAMs by modulation of these lncRNAs were demonstrated and could provide new therapeutic options in HER2+ and TNBC cases (104). In colon cancer research on macrophage-derived exosomes in M2-like macrophage polarization, their function in M2-like macrophage polarization was investigated and shown to encourage the proliferation, migration, and invasion of cancer cells. The findings recognized lncXIST as a major regulator sponging miR-17-5p, thereby boosting PDGFRA and activating significant signaling pathways like AKT, ERK, and STAT3/6. This showed how well lncRNA profiling might create more targeted medicines aimed at stopping macrophage-driven cancer spread (131). Furthermore, seen in HNSCC were exosomes formed from M1-containing lncRNA HOTTIP were discovered to control the TLR5/NF-κB signaling pathway, hence promoting cancer cell death and preventing migration. Enhanced immune response by HOTTIP-induced polarization of local and circulating macrophages to the M1 phenotype also positioned macrophage-derived lncRNAs like HOTTIP as interesting candidates for individualized cancer immunotherapy (28). These studies highlight, in general, the importance of macrophage-derived lncRNAs in cancer and support their relevance as treatment options since they can change the immune milieu to allow individualized treatment approaches.

Recent studies indicate that the best application of macrophage-derived lncRNAs to personalized oncology is through integration of immune and molecular signatures, but not as individual markers. Their practical clinical use will be based on how reproducibly they can be between patient cohorts, how they can be detected in available biospecimens, and whether they can be differentiated into signals produced by macrophages or as a response of tumor cells. Moreover, similar lncRNA can also gain unique biological importance among different types of cancers, both making it more difficult to interpret in general, but allowing immune stratification by subtype. Translational-wise, the highest utility of these molecules can be to assist in the discovery of tumors with predominantly TAM-mediated immune suppression and therefore inform patient selection to use along with checkpoint blockade, TAM reprogramming, or lncRNA-targeting intervention.

In our perspective, the clinical utility of macrophage-derived lncRNAs can be the most important since they can serve as both indicators and stratifiers at the same time. These lncRNAs could be useful in identifying precisely which tumors occur due to immune suppression through macrophages, exosomal signals or therapy-resistant microenvironmental adaptation, in contrast with conventional biomarkers, which only correlate with outcome. We thus believe that they have a high potential with accuracy oncology strategies that could be utilized to bias patients by using biomarkers and reprogram cells to be used as a macrophage or by blocking immunomodulators such as checkpoints or lncRNA. Nonetheless, such a potential will become reality as long as the future literature will be able to differentiate between true and secondary lncRNAs drivers of macrophage activation.

Altogether, macrophage-derived lncRNAs can undoubtedly serve as a personalized approach to oncology, potentially serving as markers of immune context, biomarkers of therapeutic response, and molecular targets of targeted therapy. Their clinical utility will be determined by the extent to which they can measure the macrophage-driven tumor biology in diverse cancer models and patients. This brings us to challenges and research directions in the future that are presented in the following section.

## 7 Future perspectives

Although much has been made, there is still very little development, both mechanistically and translationally, of the field of macrophage-derived lncRNAs in cancer. The fundamental issues of priority of the targets, macrophage heterogeneity, clinical validation, and therapeutic feasibility are still of great concern. This part of the paper describes significant future prospects that can bring the field to descriptive discovery to functional and clinically significant use.

One of the currently growing areas that has an enormous potential to benefit research in cancer and to provide new methods of treatment is the research on macrophage-derived lncRNAs in malignancies. Possible new directions of enhancing such lncRNAs research, their potentials in monitoring, predictions, and their broader impact on immune regulation and cancer management are outlined. To assist us in comprehending macrophage-derived lncRNAs in cancers, it is necessary to adopt modern technologies. SCRNA-seq enables a fine analysis of lncRNA patterns within macrophages, therefore, enabling the understanding of their roles in TME. Potentially appropriate genome editing strengths that the CRISPR-related functional research has identified will enable the examination of connections that exist between the lncRNA concentration and the behavior of the macrophages. In addition, the development of good *in vivo* models will be quite crucial to confirm the molecular outcome of the *in vitro* studies and assessment of the potential of treatment, prioritizing some lncRNAs. The key to speeding up advances in this science will lie with the multinational participation that incorporates bioinformatics in conjunction with molecular biology and clinical trials.

The macrophage-produced lncRNA demonstrates potential as a tumor predictive and indicative marker associated with this molecule. Their sequence expression offers opportunities in identification and tracking at an early stage because they may be indicators of cancer presence, stage, and progression. Indicatively, some lncRNAs electroponize cancer immunity, hence influencing the development and functioning of immune cells, hence controlling cancer behavior and clinical outcomes (132). Adding lncRNA analysis to the clinic would help achieve better accuracy of cancer diagnosis and provide forecasting data to guide therapy decisions.

Among the future directions, in our opinion, the redefinition of macrophage polarization in cancer by a lncRNA-centered framework instead of adhering to the traditional M1/M2 paradigm should be considered one of the highest priorities. Existing data indicate that a significant portion of tumor-associated macrophages are in hybrid/transitional phenotype, and lncRNAs can be one of the optimal regulators that maintain these intermediate phenotypes. We thus suggest that competing interests of polarization-related lncRNA circuits under spatial, temporal, and tumor contexts should be considered in future studies. This method can point to those lncRNAs that are expressed universally in macrophage reprogramming and those contexts specific and result in better mechanistic knowledge and therapeutic prioritization. Additionally, the evidence of the non-oncological models of diseases further supports the role of fundamental interactions between lncRNA and macrophage and prominently underlines its importance. Recent works by Wang et al. (2024), Sun et al. (2025), and Bian et al. (2025) indicate that lncRNAs are highly important in the regulation of macrophage activation, inflammatory signaling, and tissue remodeling in the context of cardiovascular disease, fibrosis, and sepsis (88, 133, 134). Though these studies are not cancer-specific, they offer solid mechanistic evidence in support of the idea that lncRNAs are master regulators of macrophage functional plasticity in a range of pathological environments. Such cross-disease consistency supports the hypothesis that common lncRNA-regulatory networks are active in the TME, and thus increases their usefulness as therapeutic targets in cancer.

One possible pathway in cancer immunotherapy targets macrophage-derived lncRNAs. Altering these lncRNAs might help to convert TAMs from a M2 phenotype into M1, hence improving immune responses against tumors. For example, lncRNA NORAD, produced from M2 macrophages, accelerates NSCLC growth, indicating that its suppression could have benefits in therapy (19). Combining lncRNA-targeted therapeutics with current treatments, such as immune checkpoint inhibitors, may maximize the efficiency of therapy. Still, issues like delivery systems, specificity, and possible off-target consequences need careful attention. Putting these discoveries into practical use will depend mostly on a continuous study of the function of lncRNAs in TAMs (135). Moreover, recent authenticated findings indicate that microbiota-produced metabolites have the capacity to change macrophage polarization and signaling mechanisms of immunity and indicates an extra loop of control that could be therapeutically utilized (136).

In general, we have the impression that macrophage-secreted lncRNAs can be viewed as key controllers of tumor-immune crosstalk, as opposed to incidentally relevant non-coding transcripts. The most significant addition they could make is that they can integrate environmental clues, stabilize macrophage functional programs, and propagate pro-tumor communication in the TME. In our opinion, the further development of this sphere might rely on the transition between descriptive discovery and functional prioritization, and the main focus on causal lncRNA circuits, which would regulate macrophage plasticity, immune suppression, and resist to therapy.

## 8 Conclusion

LncRNAs generated from macrophages have become increasingly important mediators of cancer development, immune regulation, and medical therapy. By inducing pro-inflammatory reactions and improving macrophage-mediated immune protection against tumors, M1-derived lncRNAs such as Cox-2 and HOTTIP show tumor-inhibitory action. By building immune-suppressive conditions, encouraging metastases, and therefore fostering treatment resistance, M2-derived lncRNAs such as AFAP1-AS1, CRNDE, and AGAP2-AS1 help to accelerate tumor advancement. HISLA and RP11-361F15.2, among TAM-derived lncRNAs, highlight even more the intricate interaction across macrophages and cancerous cells and their roles in metabolic modification, immunological escape, and angiogenesis.

In addition to their mechanical aid to tumor etiology, macrophage-derived lncRNAs have great promise as a strategy for treatment and evaluation indicators. Their potential for tumor immunotherapy is derived from their capacity to control macrophage differentiation and immunity. Targeting lncRNAs such as TUC 339, MEG3, MALAT1, and XIST, tactics have shown promise in altering macrophages toward anticancer characteristics, improving immune monitoring, and conquering treatment resistance. Nevertheless, selective delivery and accuracy of lncRNA-targeted therapy in the changing TME are still problematic. The barriers will be overcome to enable the translation of preclinical results into practical clinical therapies. Furthermore, a new cancer treatment method is demonstrated by the inclusion of lncRNAs produced by the macrophages within the personalized medicine systems. These multiple lncRNAs in the presence of several malignancies in patients and their connection to prognostic outcomes highlight their importance in personalizing certain interventions. Research that identifies some predictive lncRNAs in HCC based on ELMO1-AS1 and PTOV1-AS1, and in TNBC based on PTOV1-AS1, highlights the potential involving lncRNA-based research to manage oncology advice. Research identifying predictive lncRNAs, including ELMO1-AS1 in HCC and PTOV1-AS1 in TNBC, highlights the potential of the use of lncRNA to regulate oncology prescriptions.

Future improvements in assisting to disentangle the multiplex regulatory network orchestrated by macrophage-produced lncRNAs will include the development of single-cell RNA sequencing, CRISPR-based functional experiments, and versatile *in vivo* models. To make the best use of these molecules, bioinformatics, molecular biology, and medical research must cooperate. Macrophage-derived lncRNAs may change the landscape of cancer testing, diagnosis, and drug development, as our understanding of them expands, giving it a new way to control immunity and introduce new forms of cancer therapy.
